# Supplementary material for: Predicting the likelihood and intensity of mosquito infection from sex specific Plasmodium falciparum gametocyte density
Source: eLife. 2018 May 31;7:e34463. doi: 10.7554/eLife.34463 (PMC6013255; doi:10.7554/eLife.34463)
Supplement: Figure 1—source data 2. [file elife-34463-fig1-data2.docx]

Least squares linear regression was used to fit a best fit line for the relationship between female gametocyte density and male gametocyte density (on the logarithmic scale).

For simplicity of presentation, the model displayed in Fig 1A is a simple linear regression on all points. The slope is 0.67 (95% CI [0.60, 0.75]) and the intercept is -0.20 (95% CI [-0.46, 0.06]) and R^2^ = 0.69.

There is no evidence that a quadratic curve fit the data better than a linear relationship (p=0.815).

The fits for individual sites are given below:

| Site | Slope [95% CI] | Intercept [95% CI] | R^2^ |
| --- | --- | --- | --- |
| Ouelessebougou, Mali | 0.44 [0.32, 0.57] | 0.56 [0.02, 1.10] | 0.41 |
| Bobo Dioulasso,  Burkina Faso | 0.78 [0.40, 1.15] | 0.56 [-0.37, 1.48] | 0.53 |
| Balonghin,  Burkina Faso | 0.80 [0.67, 0.93] | -0.68 [-0.94, -0.41] | 0.79 |
| Yaoundé,  Cameroon | 0.82 [0.60, 1.04] | -0.02 [-0.87, 0.82] | 0.86 |
