# Supplementary material for: Predicting the likelihood and intensity of mosquito infection from sex specific Plasmodium falciparum gametocyte density
Source: eLife. 2018 May 31;7:e34463. doi: 10.7554/eLife.34463 (PMC6013255; doi:10.7554/eLife.34463)
Supplement: Figure 2—source data 2. [file elife-34463-fig2-data2.docx]

Infectivity was characterised as a function of gametocyte density +sing the linear, power, hyperbolic and Gompertz functions described in (1). Each curve was fit 3 times: as a function of total gametocyte density; as a function of female gametocyte density; and as a function of female and male gametocyte density (female density describing the overall shape whilst male gametocyte density allows transmission to be impeded at low densities). The full equation (extending the notation of the original manuscript) is given below,

$$g=\left[ 1-exp\left( -\tau\hat{x}_{m}' \right) \right] f_{i}\left( \hat{x}_{d}' \right)(1+ \beta_{1}y_{bobo}+\beta_{2}y_{bal}+\beta_{3}y_{cam})$$

where $\hat{x}_{d}'$ is the estimated gametocyte density, be it female parasites (*d*=*f*), male parasites (*d*=*m*) or total gametocytes (sum of females and males, *d*=*t*) and$\tau$ indicates the restriction in transmission imposed by having too few males. The function $f_{i}\left( \hat{x}_{d}' \right)$ determines the shape of the relationship between gametocytes and proportion of mosquitoes developing oocysts. Subscript *i* indicates the functional form used, be it $f_{\alpha}\left( \hat{x}_{d}' \right)=\frac{{\alpha_{1} \hat{x}_{d}'}^{\alpha_{2}}}{1+\alpha_{3} \hat{x}_{d}'}$ where constraining different parameters can generate a range of different shapes (linear $\alpha_{2}$ = 1; $\alpha_{3}$ = 0), power ($\alpha_{3}$ = 0), hyperbolic ($\alpha_{1}$> 0, $\alpha_{2}$= 1, $\alpha_{3}$> 0) or $f_{\gamma}\left( \hat{x}_{d}' \right)=\gamma_{1}\exp\left[ {-\gamma}_{2}\exp\left( -\gamma_{3}\hat{x}_{d}' \right) \right]$ which generates a Gompertz (sigmoid-like) function. The variables ybobo, ybal and ycam are indicator variables for whether a subject was from Bobo Dioulasso, Balonghin or Cameroon respectively.

All models were fit to data provided in Figure 2 – Source Data 1 using methods presented in (1) which use the dilution series trendlines to convert Ct values to estimates of gametocyte density. A full list of the models tested and their fit to data are provided below.

|  | Model fit (Deviance information criteria, DIC) | | | |
| --- | --- | --- | --- | --- |
|  | Total gametocyte density  (*d*=*t*, $\tau$=1000) | Female gametocyte density  (*d*=*f*, $\tau$=1000) | Male gametocyte density  (*d*=*m*, $\tau$=1000) | Female and male gametocyte density (*d*=*f*) |
| Linear  ($f_{\alpha}\left( \hat{x}_{d}' \right),\alpha_{2}$=1,$\alpha_{3}$=0) | 576.0 | 574.4 | 572.3 | 566.4 |
| Power  (${f_{\alpha}\left( \hat{x}_{d}' \right),\alpha}_{3}$=0) | 501.7 | 481.3 | 538.8 | **451.5** |
| Hyperbolic  ($f_{\alpha}\left( \hat{x}_{d}' \right), \alpha_{2}$=1) | 573.0 | 564.8 | 517.4 | + |
| Gompertz  ($f_{\gamma}\left( \hat{x}_{d}' \right)$) | 557.4 | 555.7 | 564.0 | 548.3 |

+ models with this combination of functions failed to converge and so were discounted.

The model with the lowest DIC is the power function as a function of Female gametocyte density multiplied by (*1*-exp(-*a*g_m_*)) is presented in Figure 2A. Estimates and credible intervals for the coefficients are given in the table below:

| Coefficient | Estimate | 95% credible interval |
| --- | --- | --- |
| $\tau$ | 0.08573 | (0.04229, 0.1967) |
| $\alpha_{1}$ | 0.07108 | (0.02295, 0.1746) |
| $\alpha_{2}$ | 0.3021 | (0.1602, 0.4751) |
| $\beta_{1}$ | 2.65 | (1.273, 5.174) |
| $\beta_{2}$ | -0.04733 | (-0.9017, 1.405) |
| $\beta_{3}$ | 0.6757 | (0.06286, 1.582) |

*Microscopy data*

The performance of models including both qRT-PCR and microscopy in the same model are investigated. This is to determine whether there is value in combining gametocyte density estimates collected with different methodologies as additional information may be added by the less precise diagnostic. These data could be combined using a fully Bayesian method, taking into account the number of gametocytes counted and the quantity of blood examined. However, these non-standard analytical methods are unlikely to be used in field or laboratory settings so combined density estimates, generated by both qRT-PCR and microscopy, are calculated by taking the mean of both estimates (i.e. assuming both diagnostics have equal weight for the overall estimate).

The methods outlined in the main text allows the uncertainty in the qRT-PCR estimates to be incorporated into a Bayesian model. Only point estimates are availible for microscopy so the models are fit using using least squares methodology and compared with the R-squared (*R^2^*) statistic. Microscopy detects both female and male gametocytes (though they are not easy to sex) so microscopy data is combined with total gametocyte density by qRT-PCR. The power function was used for all models reflecting the best fit of the qRT-PCR outlined above.

The amount of variarion explained by each of the models is outlined below. Model accuracy was similar in all models, altough qRT-PCR methods performed slightly better than microscopy alone or when it was combined with qRT-PCR.

| **Oocyst prevalence dependent on:** | ***R^2^*** |
| --- | --- |
| Total gametocyte density by PCR | 0.71 |
| Total gametocyte density by microscopy | 0.61 |
| Mean of total gametocyte density by PCR and total gametocyte density by microscopy | 0.67 |

1. Churcher TS, Bousema T, Walker M, Drakeley C, Schneider P, Ouedraogo AL, et al. Predicting mosquito infection from Plasmodium falciparum gametocyte density and estimating the reservoir of infection. Elife. 2013;2:e00626.
